# Secondary prevention lifestyle interventions initiated within 90 days after TIA or ‘minor’ stroke: a systematic review and meta-analysis of rehabilitation programmes

**DOI:** 10.3399/bjgp16X688369

**Published:** 2016-12-06

**Authors:** Neil Heron, Frank Kee, Christopher Cardwell, Mark A Tully, Michael Donnelly, Margaret E Cupples

**Affiliations:** Centre for Public Health, School of Medicine, Dentistry and Biomedical Science, Queen’s University Belfast; UKCRC Centre of Excellence for Public Health (Northern Ireland), Institute of Clinical Science B, Royal Victoria Hospital, Belfast.; Centre for Public Health, School of Medicine, Dentistry and Biomedical Science, Queen’s University Belfast; UKCRC Centre of Excellence for Public Health (Northern Ireland), Institute of Clinical Science B, Royal Victoria Hospital, Belfast.; Centre for Public Health, School of Medicine, Dentistry and Biomedical Science, Queen’s University Belfast; UKCRC Centre of Excellence for Public Health (Northern Ireland), Institute of Clinical Science B, Royal Victoria Hospital, Belfast.; Centre for Public Health, School of Medicine, Dentistry and Biomedical Science, Queen’s University Belfast; UKCRC Centre of Excellence for Public Health (Northern Ireland), Institute of Clinical Science B, Royal Victoria Hospital, Belfast.; Centre for Public Health, School of Medicine, Dentistry and Biomedical Science, Queen’s University Belfast; UKCRC Centre of Excellence for Public Health (Northern Ireland), Institute of Clinical Science B, Royal Victoria Hospital, Belfast.; Centre for Public Health, School of Medicine, Dentistry and Biomedical Science, Queen’s University Belfast; UKCRC Centre of Excellence for Public Health (Northern Ireland), Institute of Clinical Science B, Royal Victoria Hospital, Belfast.

**Keywords:** behaviour change techniques, early rehabilitation, lifestyle interventions, ‘minor’ stroke, secondary prevention, transient ischaemic attack

## Abstract

**Background:**

Strokes are often preceded by a transient ischaemic attack (TIA) or ‘minor’ stroke. The immediate period after a TIA/minor stroke is a crucial time to initiate secondary prevention. However, the optimal approach to prevention, including non-pharmacological measures, after TIA is not clear.

**Aim:**

To systematically review evidence about the effectiveness of delivering secondary prevention, with lifestyle interventions, in comprehensive rehabilitation programmes, initiated within 90 days of a TIA/minor stroke. Also, to categorise the specific behaviour change techniques used.

**Design and setting:**

The review identified randomised controlled trials by searching the Cochrane Library, Ovid MEDLINE, Ovid EMBASE, Web of Science, EBSCO CINAHL and Ovid PsycINFO.

**Method:**

Two review authors independently screened titles and abstracts for eligibility (programmes initiated within 90 days of event; outcomes reported for TIA/minor stroke) and extracted relevant data from appraised studies; a meta-analysis was used to synthesise the results.

**Results:**

A total of 31 potentially eligible papers were identified and four studies, comprising 774 patients post-TIA or minor stroke, met the inclusion criteria; two had poor methodological quality. Individual studies reported increased aerobic capacity but meta-analysis found no significant change in resting and peak systolic blood pressure, resting heart rate, aerobic capacity, falls, or mortality. The main behaviour change techniques were goal setting and instructions about how to perform given behaviours.

**Conclusion:**

There is limited evidence of the effectiveness of early post-TIA rehabilitation programmes with preventive lifestyle interventions. Further robust randomised controlled trials of comprehensive rehabilitation programmes that promote secondary prevention and lifestyle modification immediately after a TIA are needed.

## INTRODUCTION

Stroke killed 5.7 million people worldwide in 2005 and was expected to cause around 6.5 million deaths in 2015.^1^ Survivors of stroke often suffer considerable residual disability.^2^ Many strokes are preceded by transient ischaemic attacks (TIAs) in the previous 90 days,^3^ and therefore the immediate period after a TIA is a crucial time to intervene to try to counter known vascular risk factors and reduce the risk of subsequent stroke.

The 90-day risk of vascular events following a TIA or ‘minor’ stroke, excluding events within the first week after diagnosis when the risk is highest, can be as high as 18%.^4^ The presence of a new infarct (identified via brain imaging and indicating that a patient has had a stroke rather than a TIA) places a patient at higher risk of a further stroke within the first 90 days.^5^ However, an immediate assessment following the initial event and the initiation of secondary prevention that is focused mainly on pharmacological interventions may reduce the 90-day risk of stroke to 2% within a research setting,^6^ although these results have not been replicated in routine practice.^7^ There is a need for clearer guidance regarding optimal early prevention and rehabilitation strategies and interventions to promote lifestyle change following TIA. Some current guidelines^8^^,^^9^ advocate the promotion of early non-pharmacological secondary prevention after all cerebrovascular events but lack detail regarding effective methods of its delivery. There have been reports of lifestyle interventions post-TIA and minor stroke.^10^^–^12 However, the authors have not identified any previous systematic reviews of the evidence relating to the effectiveness of early post-TIA rehabilitation or secondary prevention programmes that describe lifestyle interventions.

While patients who have experienced TIA and minor stroke are without residual physical functional disability, they often have residual functional impairment, particularly with regard to post-event fatigue and psychological issues, which may lead to more significant disability.^13^^,^^14^ However, these issues are not currently highlighted in early rehabilitation or secondary prevention programmes for patients with TIA/minor stroke, although the novel adaptation of cardiac rehabilitation programmes for patients who have experienced TIA and stroke has been advocated.^15^^–^17 Comprehensive programmes, which try to alter participants’ behaviours, are complex: information about their ‘active’ ingredients, such as specific behaviour change techniques,^18^ would facilitate their replication and the implementation of guidelines for good clinical practice.^19^^,^^20^

This review aimed to investigate the effect of comprehensive rehabilitation programmes which included secondary prevention lifestyle measures, initiated within 90 days of a TIA or ‘minor’ stroke in adults;^20^^,^^21^ and to identify and categorise the behaviour change techniques that were employed in these programmes.

**How this fits in**

Although immediate post-transient ischaemic attack (TIA) and/or minor stroke secondary prevention lifestyle programmes are theoretically of value, few studies have been conducted in this area. The level of evidence available for the effectiveness of early implementation of lifestyle intervention programmes following TIA or minor stroke is poor. There is a clear need for further robust randomised controlled trials of lifestyle intervention programmes that promote secondary prevention during the acute post-TIA period.

## METHOD

This systematic review is reported in line with the Preferred Reporting Items for Systematic Reviews and Meta-analyses (PRISMA) guidance,^22^ and has been reported as per previous authors.^23^ Human randomised and quasi-randomised controlled trials of comprehensive rehabilitation programmes initiated within 90 days of patients suffering a TIA or minor stroke were included.

The review focused on adults, males and females, aged ≥18 years, who received a diagnosis of a TIA and/or ‘minor’ stroke, based on clinical diagnosis, or on findings from brain imaging (for example, computed tomography or magnetic resonance imaging of the head). Studies were excluded which included only patients experiencing moderate or severe stroke, which did not provide outcome data specifically for patients with TIA/minor stroke, and in which the intervention was initiated later than 90 days post-event.

Any comprehensive rehabilitation programme, identified as a set of measures to achieve and maintain a patient’s optimal functioning,^24^ following an initial TIA or ‘minor’ stroke, was eligible for inclusion. The review included any one-to-one or group-based intervention that was undertaken in hospital, outpatient department, community, or the home. Trials with a comparative control group and trials with multiple intervention arms (comparing different forms of rehabilitation) were included. The review did not include population or community-wide interventions.

### Types of outcome measures

#### Primary outcomes

These were quantitative between-group differences for modifiable risk factors (for example, blood pressure, lipid and triglyceride profiles, markers of insulin resistance and obesity, validated cardiovascular risk score, and tobacco use) or level of functioning and/or disability, including social and emotional functioning.

#### Secondary outcomes

Secondary cardiovascular events were: stroke, myocardial infarction, or vascular death; any adverse events such as exercise-related musculoskeletal injuries; or any indicator of patient adherence to secondary prevention medication such as self-reported medication adherence.

### Search methods for identification of studies

Detailed search strategies were developed for each electronic database searched with input from a medical librarian to allow identification of studies for inclusion in this review. The searches were based on the strategy developed for Medical Literature Analysis and Retrieval System Online (MEDLINE) (details available from the authors on request) but revised appropriately for each database. Searches were carried out using the following databases: the Cochrane Library, Ovid MEDLINE 1946 to March 2015, Ovid Embase 1974 to March 2015, Web of Science, EBSCO Cumulative Index to Nursing and Allied Health Literature (CINAHL) plus 1937 to March 2015, and Ovid PsycINFO 1806 to March 2015. Any systematic reviews of rehabilitation interventions in the acute period following a TIA or ‘minor’ stroke were screened for additional references. The titles and abstracts of publications from the search strategy were independently screened by two authors. Additional studies were identified from reviewing the reference lists of the retrieved papers through a hand search. Articles not meeting the inclusion criteria were discarded. A standardised form (details available from the authors on request) was used to select the trials eligible for inclusion in the review with, if necessary, a third review author resolving disagreements. A record was kept of all articles excluded at this stage and the reason for their exclusion. No language restrictions were made, although all papers were written in the English language.

Data on methodological issues, eligibility criteria, interventions (including the number of participants treated and intervention provider), and study design, study duration, follow-up, comparisons, outcome measures, results, withdrawals, and adverse events were extracted independently by two review authors. There was no blinding to study author, institution, or journal, and a record was kept of each study included in the review.

### Assessment of quality and risk of bias

The PEDro scale^25^ was used to assess the quality of included papers in the review. Also, two review authors independently assessed each included study for risk of bias (‘high’, ‘low’, or ‘uncertain’) using the risk of bias tool, following guidance from the *Cochrane Handbook for Systematic Reviews of Interventions*,^26^ with a third review author acting as arbitrator as required.

### Measures of treatment effect

For each study, relative risk and 95% confidence intervals (CIs) were calculated for dichotomous outcomes and mean differences, and 95% CIs were calculated for continuous outcomes. Where continuous outcomes were pooled on different scales, standardised mean differences were used. Where available, changes from baseline (mean change scores) were used in preference to follow-up scores. When combining results for the individual studies, mean differences and a random effects model were used apart from two variables, deaths and falls, when odds ratios with a random effects model were used.

### Assessing for heterogeneity

Diversity across the studies was assessed qualitatively in terms of intervention (content, duration, frequency, provider, and setting), participant demographics, outcome measures, and follow-up. If two or more studies were considered clinically homogenous according to the above terms, data were assessed for statistical heterogeneity using RevMan (version 5.1). The χ^2^ test was used in conjunction with the *I*^2^ statistic, which describes the percentage of variability in effect estimates due to heterogeneity. The level of significance for the χ^2^ test was set at *P*<0.1. Values of *I*^2^ from 30% to 60% were considered to represent moderate heterogeneity and 60% to 90% substantial heterogeneity.^26^

### Data synthesis

Careful consideration was given to the appropriateness of conducting a meta-analysis. Data were summarised statistically when the data were available, sufficiently similar, and of sufficient quality, and the statistical analysis was performed in accordance with guidelines.^26^

### Behaviour change techniques

Two trained review authors independently screened studies included in the review for the reported use of behaviour change techniques. Michie’s behaviour change technique taxonomy,^18^ comprising 93 hierarchically clustered techniques, was used to identify the behaviour change techniques, and a narrative approach was used to describe their use in the rehabilitation programmes.

## RESULTS

**Figure 1.**
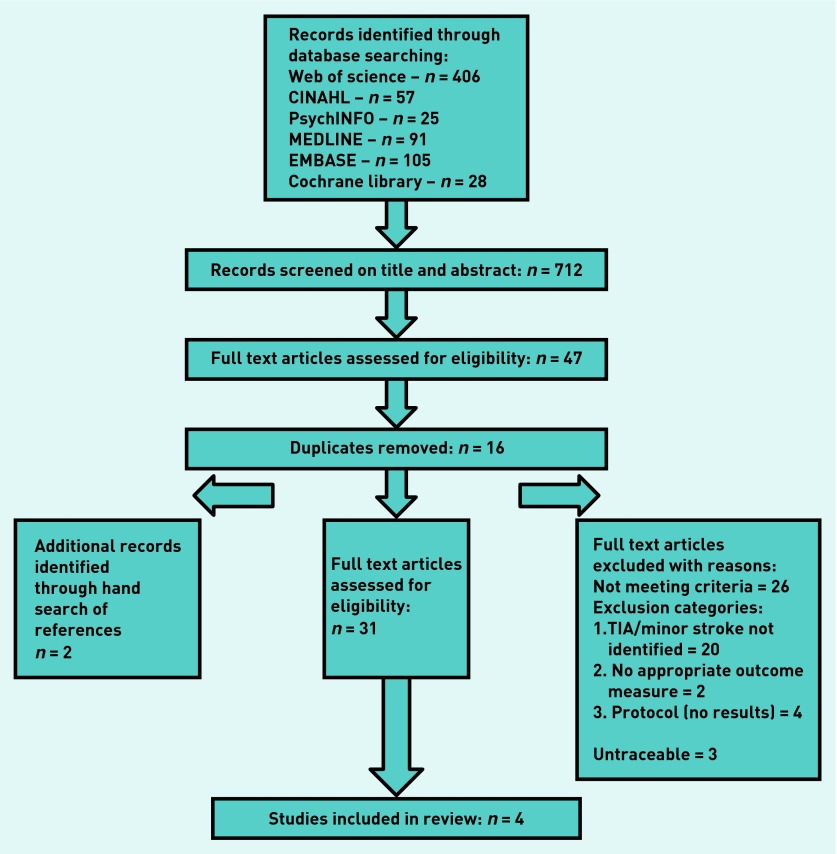
***Flow diagram of reviewed and included papers. TIA = transient ischaemic attack.***

The full text of 47 studies was reviewed and 31 studies were identified for possible inclusion in this review (Figure 1). Only two of the 31 studies were included and an additional two studies were retrieved from the reference lists of the previously identified 31 studies. Three studies could not be traced. Fourteen studies were excluded because the sample comprised participants with moderate or severe stroke, and six studies were excluded because they included participants with varied stroke severity and did not provide outcome data for patients with TIA. A further six papers were excluded because they were protocols (*n* = 2), editorials/overviews (*n* = 2), or did not have appropriate outcome measures (*n* = 2).

### Programme design and evaluation

Four studies were included in the review (Table 1). Allen and colleagues^27^ evaluated whether comprehensive post-discharge care management for 380 survivors of minor stroke was superior to organised acute stroke department care with enhanced discharge planning. Toledano-Zarhi *et al* 28 examined the feasibility, safety, and effectiveness of an early aerobic rehabilitation programme for 28 patients between 1 and 3 weeks post-minor ischaemic stroke. Boysen and colleagues^29^ investigated the effect of repeated verbal instructions about physical activity delivered to 314 patients within 90 days of a minor ischaemic stroke, 3-monthly for the first year and 6-monthly for a second year. Tanne *et al* 30 assessed the tolerability, safety, and effect of an outpatient supervised exercise training programme, in 52 patients after a non-disabling stroke, initiated within a mean time of 65 days. Two of the included studies were conducted in Israel,^28^^,^^30^ one in the US,^27^ and one in China and Europe (Denmark, Poland, and Estonia).^29^ All patients had a diagnosis of TIA and/or ‘minor’ ischaemic stroke, and were recruited from secondary care, typically from stroke units. Three studies delivered the intervention in secondary care,^28^^–^30 while one was delivered at home by a multidisciplinary team after an initial consultation in hospital.^27^ Outcomes were assessed after 6 weeks,^28^ 3 months,^30^ 6 months,^27^ and 2 years.^29^

**Table 1.** Information on included studies, risk of bias and PEDro Score

**Study**	**Sample size**	**Dropouts**	**Intervention design**	**Primary outcome measure**	**Secondary outcome measure(s)**	**Follow-up duration**	**Control**	**Risk of bias**	**PEDro score**
Allen *et al* 27	Intervention (I) 190; Control (C) 190	I: 25 C: 36	Comprehensive post-discharge care management intervention; assessment by nurse in participant’s home; reviewed by the treating medical team. Patient care plans developed. Periodic telephone calls to assess change	None stated	Neuromotor function (measured using NIHSS, Timed Up and Go test, and physical performance test); institution time (days spent hospitalised or in a nursing home during 6-month follow-up); death; quality of life (stroke-specific QOL scale); systolic and diastolic blood pressure (mmHg), depression (CES-D scale), medication appropriateness (an investigator generated tool), haemoglobin A1c (%), total cholesterol (mg/dL), self-reported falls and incontinence; stroke knowledge and lifestyle modification (an investigator-generated questionnaire that assesses knowledge of stroke risk factors and health behaviours)	6 months	Usual post-discharge care planning	Low	9
Tanne *et al* 30	I: 43 C: 9	I: 2 C: 2	Education on vascular risk, physical exercise and healthy lifestyle; supervised exercise programme, twice/week for 3 months (15 minutes warm-up, 45 minutes on treadmill, stair machine and bicycle at 60–70% of maximal heart rate); prescribed by physiologist; supervised by physical therapy and cardiac rehabilitation staff. Exercise prescription adjusted if capacity improved	Physical fitness — maximal exercise test, 6-minute walk test (metres walked)	Resting heart rate (BPM) and resting systolic blood pressure (mmHg)	3 months	Usual post-TIA/stroke care	High. Pilot non-random trial	6
Toledano-Zarhi *et al* 28	I:14 C: 14	I: 1 C: 0	Exercise group enrolled in 6-week supervised exercise programme (3 hours weekly: 2 sessions of 35–55 minutes on treadmill, hand-bike, and bicycle, supervised by physical therapy and cardiac rehabilitation staff: 8 progressive stages; also, 45–55 minutes group practice for strength, flexibility, and coordination Exercise prescription adjusted if capacity improved	Exercise capacity — maximal exercise test, 6-minute walk test (metres walked)	Adverse events (for example strokes or falls)	6 weeks	Home-exercise booklet, advising strength and flexibility exercises, plus normal routine	Uncertain	7
Boysen *et al* 29	I: 157 C: 157	I: 24 C: 14	Repeated encouragement and verbal instruction on being physically active given by a physiotherapist or neurologist	Physical activity assessed with the Physical Activity Scale for the Elderly	Clinical events, for example number of strokes, or hospitalisations	2 years	Verbal information on benefits of physical activity	Low risk	9

BPM = beats per minute. CES-D = Center for Epidemiologic Studies - Depression. NIHSS = National Institutes of Health Stroke Scale. PEDro = Physiotherapy Evidence Database. QOL = quality of life. TIA = transient ischaemic attack.

### Behavioural change techniques in included studies

The behaviour change technique identified as ‘instruction on how to perform a behaviour’ was employed in all four studies. ‘Goal setting’ was used in three of the four studies, and a range of other behaviour change techniques were used by different studies (Box 1). One outpatient exercise programme,^30^ a feasibility study, was the only programme that used the behaviour change technique of ‘discussing behavioural consequences’ with patients, and this study reported a statistically significant improvement in post-event exercise capacity.

Box 1. **Behaviour change techniques used by the included studies**

**Table:** 

**BCT label**	**BCT group**	**Example of how the BCT was used (reference)**	**Studies using BCT (*n*: references)**
Instruction on how to perform a behaviour	Shaping knowledge	*‘... education regarding lifestyle modification’* 27	4: 27, 28, 30, 29
Goal setting (behaviour)	Goals and planning	*‘… exercise at “60–70% of maximal heart rate, as prescribed by a physiologist”.’* 30	3: 28, 30, 29
Action planning	Goals and planning	*‘... twice-weekly session of 35–55 minutes on a treadmill, a hand-bike machine and a bicycle … A pulse rate target of 50–70% of maximal heart rate’* 28	2: 28, 29
Credible source	Comparison of outcomes	*‘The experimental intervention consisted of repeated encouragement and verbal instruction on being physically active given by a physiotheraptist’* 29	2: 27, 29
Monitoring of behaviour by others without feedback	Feedback and monitoring	*‘Compliance was monitored’* 28	2: 27, 28
Review behaviour goal(s)	Goals and planning	‘*Participants were ‘“encouraged to increase efforts and to exercise more”.’* 29	1: 29
Behavioural contract	Goals and planning	*‘… instructor and participants would fill in a standard agreement form with various choices of physical activity’* 29	1: 29
Self-monitoring of behaviour	Feedback and monitoring	*‘... received a personalised health record to help them self-manage their risk factors’* 27	1: 27
Social support (unspecified)	Social support	*‘Considerable effort was taken to motivate the participants’* 29	1: 29
Social support (practical)	Social support	*‘… ensure that needed social services (for example Meals on Wheels) were in place’* 27	1: 27
Social support (emotional)	Social support	*‘… intervention to reduce common post-stroke complications (for example, depression)’* 27	1: 27
Information about health consequences	Natural consequences	*‘... educated on vascular risk factors and the importance of physical exercise’* 30	1: 30
Demonstration of the behaviour	Comparison of behaviour	*‘… group practice for inducing strength, flexibility, and coordination performances’* 28	1: 28
Prompts/cues	Associations	*‘… repeated instructions and readjustment of the physical activity plan’* 29	1: 29
Behavioural practice/rehearsal	Repetition and substitution	*‘… group practice for inducing strength, flexibility, and coordination performances was performed once a week’* 28	1: 28
Pharmacological support	Regulation	*‘... medication reconciliation and pill organisers to optimise stroke risk factor control’* 27	1: 27
Adding objects to the environment	Antecedents	*‘… and pill organisers to optimise stroke risk factor control’* 27	1: 27

*BCT* = *behaviour change technique.*

Within the behaviour change technique taxonomy,^18^ individual behaviour change techniques are clustered into hierarchical groups that commonly appear together in behavioural interventions. The commonest group of behaviour change techniques used in the four included studies was ‘goals and planning’, while the second most common group used was ‘sharing knowledge’. Of all the groups of behaviour change techniques listed in the taxonomy,^18^ five were not reported as having been used in any of the studies: ‘reward and threat’, ‘antecedent identity’, ‘scheduled consequences’, ‘self-belief’, and ‘covert learning’.

Regarding use of the behaviour change technique ‘instruction on how to perform a behaviour’, one study reported that a physiotherapist or neurologist provided ‘repeated encouragement and verbal instructions on being physically active’.^29^ Toledano-Zarhi and colleagues^28^ reported that they provided all participants with a home exercise booklet, including information on strength and flexibility exercises, as well as encouraging them to maintain their normal physical activity routine in the community. The participants in the active group then additionally received a supervised exercise programme twice weekly for 3 hours a week over a duration of 6 weeks. Meanwhile in the study by Tanne *et al* 30 the intervention group were entered into a supervised exercise training programme performed twice weekly over a 3-month duration. The exercise programme was prescribed by an exercise physiologist, with specific guidance regarding intensity, type, and duration of exercise. Allen and colleagues^27^ reported that they offered general lifestyle modification advice to address secondary prevention factors.

### Risk of bias in included studies

The studies by Allen *et al* 27 and Boysen *et al* 29 were deemed to be at low risk of bias, while that of Tanne and colleagues^30^ was assessed as being at high risk of bias because it was a pilot, quasi-randomised controlled trial (Table 1). The study by Toledano-Zahri *et al* 28 was assessed as being at uncertain risk of bias because there was no detail reported regarding randomisation or blinding of participants and assessors. The application of the PEDro scale^25^ supported these assessments. Allen and colleagues^27^ and Boysen *et al* 29 had allocation concealment within their study design, thus reducing their risk of bias, in contrast to studies by Tanne and colleagues^30^ and Toledano-Zarhi *et al.*^28^ The study by Allen and colleagues 27 is unclear regarding blinding of participants and personnel for study allocation but outcome measurements were blinded. Tanne *et al* 30 and Toledano-Zarhi and colleagues^28^ did not detail how their groups were allocated or if personnel were blinded to study allocation. Boysen *et al* 29 described how personnel undertaking their outcome assessment, the Physical Activity Scale for the Elderly, were blinded to group allocation. The proportion of study participants completing follow-up ranged from 92% in the intervention arm,^29^ to 100%.^27^^,^^28^ All studies fully accounted for the study participants and provided reasons for any missing data. No other potential sources of bias were identified.

### Assessment of reporting bias

Funnel plots were not produced because only two studies were included in each of the outcome measures.

### Effects of interventions

One study reported the percentage dead at end of study, percentage smoking, and percentage exercising;^27^ one reported change in the Physical Activity Scale for the Elderly, number of vascular events, and change in modified Rankin Scale;^29^ and two shared similar outcome measures: 6-minute walk test, exercise test results (metabolic equivalents achieved), and resting and peak blood pressure.^28^^,^^30^ There was a high risk of bias in Tanne *et al’*s work,^30^ and uncertain risk of bias in the study by Toledano-Zahri and colleagues^28^ but their results were included in a meta-analysis, to explore potential insights, while being cautious about interpretation. A sensitivity analysis was not undertaken because the results of the meta-analysis were non-significant.

**Figure 2.**
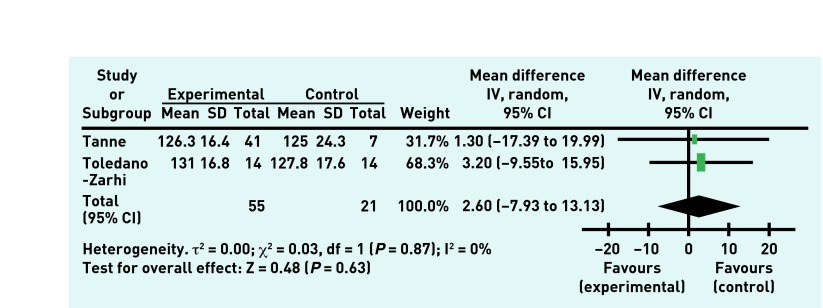
***Resting systolic blood pressure (mmHg) in experimental and control groups post-intervention.***

**Figure 3.**
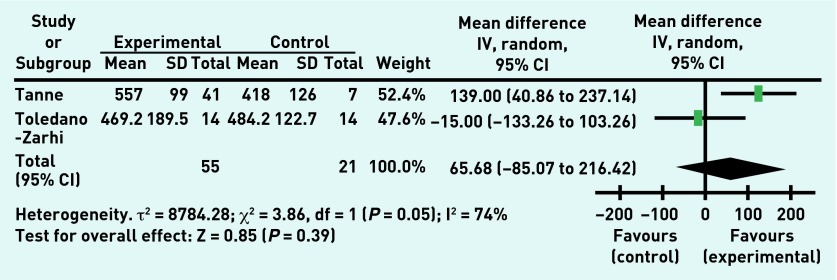
***Six-minute walking test performance (metres walked) in experimental and control groups post-intervention.***

**Figure 4.**
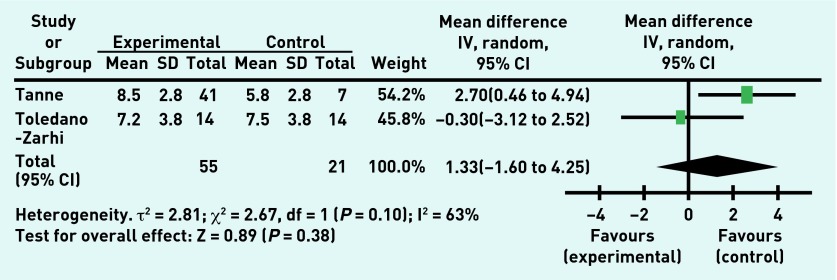
***Exercise testing result in experimental and control groups post-intervention.***

The overall treatment effect showed no improvement in the resting systolic blood pressure (Figure 2) or in peak systolic blood pressure. There was no treatment effect for resting heart rate nor for the 6-minute walk test (Figure 3), although improvement in the 6-minute walk test was shown by one study, a 3-month outpatient exercise programme.^30^ The overall treatment effect showed no improvement in the exercise testing result (Figure 4), although one study did show improvement in the exercise test results.^30^ There was no effect shown on the number of falls experienced by the participants during the rehabilitation programme or in rates of death following the rehabilitation programme. Of note, two studies^28^^,^^30^ reported no deaths during their studies and so these results were unable to be incorporated into the meta-analysis.

## DISCUSSION

### Summary

This systematic review comprised four studies which initiated comprehensive rehabilitation programmes, including lifestyle and pharmacological interventions, within 90 days of a diagnosis of TIA or ‘minor’ stroke; although only two studies were of high methodological quality. Many other potentially eligible studies were excluded from the review because they did not present results specifically for patients who had experienced TIA or minor stroke, the focus of previous research being on moderate or severe stroke secondary prevention and rehabilitation. A meta-analysis found no evidence of significant effects on subsequent stroke risk or on vascular risk factors, although two studies reported improved measures of exercise capacity.^28^^,^^30^

This review, the first to report collated observations on the behaviour change techniques used in studies of comprehensive rehabilitation programmes for patients with acute TIA or minor stroke, suggests that providing individualised instruction to patients about how to perform specific behaviours and behavioural goal setting are key programme components but many other behaviour change techniques have been used. One programme, which reported improved exercise capacity,^30^ was the only one that included discussion of behavioural consequences with patients, hinting at the importance of using this behaviour change technique in future programmes. As the emphasis on early recognition of stroke and TIA in clinical practice increases, it is important to know more about appropriate comprehensive management programmes for these patients who may not have obvious residual disability and whose needs for non-pharmacological support and rehabilitation may be overlooked.

### Strengths and limitations

This review attempted to identify all studies of potential relevance by developing a comprehensive search strategy and then supporting this through hand searching reference lists of all the full text articles examined in the review. The authors sought to include all eligible studies regardless of publication language, although all studies were in English.

This systematic review and meta-analysis is limited by the few studies available in this expanding and novel area of research: only four met the inclusion criteria. The authors acknowledge that the search strategy focused on ‘rehabilitation’ rather than secondary prevention programmes, so may have missed potentially relevant studies. However, this has served to highlight the lack of research focus on rehabilitation or early initiation of lifestyle interventions in secondary prevention for patients with TIA. The most common reasons for exclusion of potentially eligible studies from this review were that the studies included moderate and severe strokes, with no focus on TIA/minor stroke or outcomes specified for these patients, and that the rehabilitation programme was not initiated in the acute period following diagnosis. If data were available for the patients experiencing TIA and minor stroke in studies that were excluded because they did not differentiate stroke severity for their participants, further meta-analysis could be performed to add to the strength of evidence available.

A limitation of the included studies was the lack of consistency in the outcome measures used and combining results in a meta-analysis was therefore difficult. Another study limitation related to the duration of follow-up, which varied from 6 weeks to 2 years. Data collected were pooled at the end of the study as per protocol. The results should therefore be interpreted with caution because shorter study durations may not allow sufficient time for the rehabilitation interventions to produce an impact on modifiable vascular risk factors.

Intervention intensity is generally a poorly defined concept,^31^ and differences in this are considered to be a source of heterogeneity within complex interventions.^31^ Intervention intensity was different across the included studies and could be a potential source of bias within the review.

Further, while patients with TIA often have residual functional impairment, with fatigue and psychological issues,^13^^,^^14^ there was little recognition of this within the outcome measures used in the studies reviewed.

### Comparison with previous literature

Evidence is growing regarding the contribution of change in modifiable risk factors to reductions in cardiovascular deaths,^32^ and there is a need to consider how to promote non-pharmacological measures in secondary prevention.^33^ Indeed, five modifiable risk factors are reported to account for 82% of strokes: hypertension, smoking, obesity, unhealthy diet, and physical inactivity.^34^ A pilot study of a community-based cardiac rehabilitation post-TIA and ‘mild’ stroke, for patients up to 1 year post-event, showed reductions in biological markers linked to cardiovascular and cerebrovascular mortality, including aerobic capacity, lipid profile, waist circumference, body mass index, body weight, and smoking status at 6 months, but did not include a control group.^16^ Similarly, Kirk and colleagues^17^ found that community-based cardiac rehabilitation programmes for 12 patients post-TIA and minor stroke can be effective at reducing modifiable vascular risk factors. Reviews identifying effective components of rehabilitation following the diagnosis of a TIA and ‘minor’ stroke have begun to emerge,^10^^–^12^,^^31^ but, to the authors’ knowledge, this is the first systematic review of the use of comprehensive rehabilitation programmes in the acute period (within 90 days) of the diagnosis of a TIA or ‘minor’ stroke.

No significant effect on vascular risk factors was found in this review. However, Brook *et al* 35 reviewed non-pharmacological treatment effects on blood pressure and found Class 1, level A evidence for the blood pressure-lowering effects of exercise in patients with cardiovascular disease. Billinger and colleagues^36^ performed an 8-week exercise programme in 10 subjects with moderate severity stroke in the sub-acute period following their diagnosis, with a reduction in resting heart rate and improvement in the 6-minute walk test performance post-intervention. Mackay-Lyons and Makrides^37^ showed no change in peak systolic blood pressure when following survivors of stroke, undifferentiated by severity, for 6 months despite improvements in aerobic capacity. Duncan *et al* 38 found that an 8-week home-based therapist-supervised exercise programme undertaken three times a week within 30 to 90 days of a mild-to-moderate stroke improved 6-minute walk test performance in the intervention group. Thus, while previous work shows improvements in vascular risk factors associated with exercise for survivors of stroke, this review highlights the lack of evidence of this effect following rehabilitation programmes initiated early after a TIA and/or ‘minor’ stroke. It is possible that patients who entered these studies have been given optimal pharmacological management and that there is limited opportunity to show statistically significant differences between groups but it was noted that none of the studies included in this review were powered to detect changes in blood pressure. This is clearly an important area that requires urgent further research.

No significant reduction in the number of falls was found in this meta-analysis. However, Taylor-Piliae and colleagues^39^ found that exercise, particularly Tai Chi-based programmes, improved balance and reduced falls in patients aged >50 years, who had suffered a stroke up to 3 years previously. A 12-week supervised exercise programme in survivors of sub-acute stroke also demonstrated improvements in balance.^40^ However, a Cochrane Review^41^ has shown no consistent evidence for exercise programmes preventing falls in survivors of stroke. Thus the evidence is mixed in relation to falls reduction and further high-quality studies are required in this area.

No excess deaths were found in those undergoing rehabilitation programmes compared with those not undergoing rehabilitation and thus acute rehabilitation programmes appear safe in this patient cohort. Indeed, exercise guidelines for survivors of stroke have been established, with exercise in this cohort of patients considered to be safe.^42^

The National Institute for Health and Care Excellence published guidance in 2014 on individual-level behaviour change interventions for promoting change in modifiable cardiovascular risk factors.^20^ These guidelines recommended that behaviour change programmes, including lifestyle management programmes, should include behaviour change techniques relating to goals and planning, feedback and monitoring, and social support. In keeping with these guidelines,^20^ the most frequent behaviour change techniques found in this review were facilitating patients to set goals for behavioural changes and providing instruction on how to perform the new behaviours. However, the provision of social support was only implemented in two of the four studies,^27^^,^^29^ These specific behaviour change techniques represent only a small fraction of those available in Michie’s taxonomy, so that there is clear scope for further research. This review highlights the poor reporting of behaviour change techniques used in interventions. It is clearly important to identify the specific behaviour change techniques used in studies to allow their replication. Future studies of rehabilitation and secondary prevention following a TIA and ‘minor’ stroke should develop descriptions of the use of these techniques within their interventions.

### Implications for research and practice

Few studies have reported the effects of early initiation of comprehensive rehabilitation programmes, with or without pharmacological interventions, on secondary prevention for patients who have experienced a TIA or ‘minor’ stroke. Rather, the focus of previous work has been on patients having ‘moderate’ and ‘severe’ strokes. Gaps have been identified in the knowledge regarding the effectiveness of comprehensive rehabilitation programmes, that include pharmacological and lifestyle measures, initiated in the acute period following a TIA or ‘minor’ stroke when the risk of further vascular events is highest. Further randomised controlled trials, which include lifestyle interventions and details regarding the behaviour change techniques used for these patients are needed. In particular, further studies should be carried out to determine the value of initiating comprehensive secondary prevention, including non-pharmacological management, in the acute period following a TIA/minor stroke. One such emerging study is SPRITE,^43^ which aims to examine the effectiveness of a home-based rehabilitation programme, promoting the role of primary care in managing cardiovascular risk factors in the acute period following a TIA/minor stroke, particularly via non-pharmacological methods, for example, smoking cessation advice and physical activity promotion.
